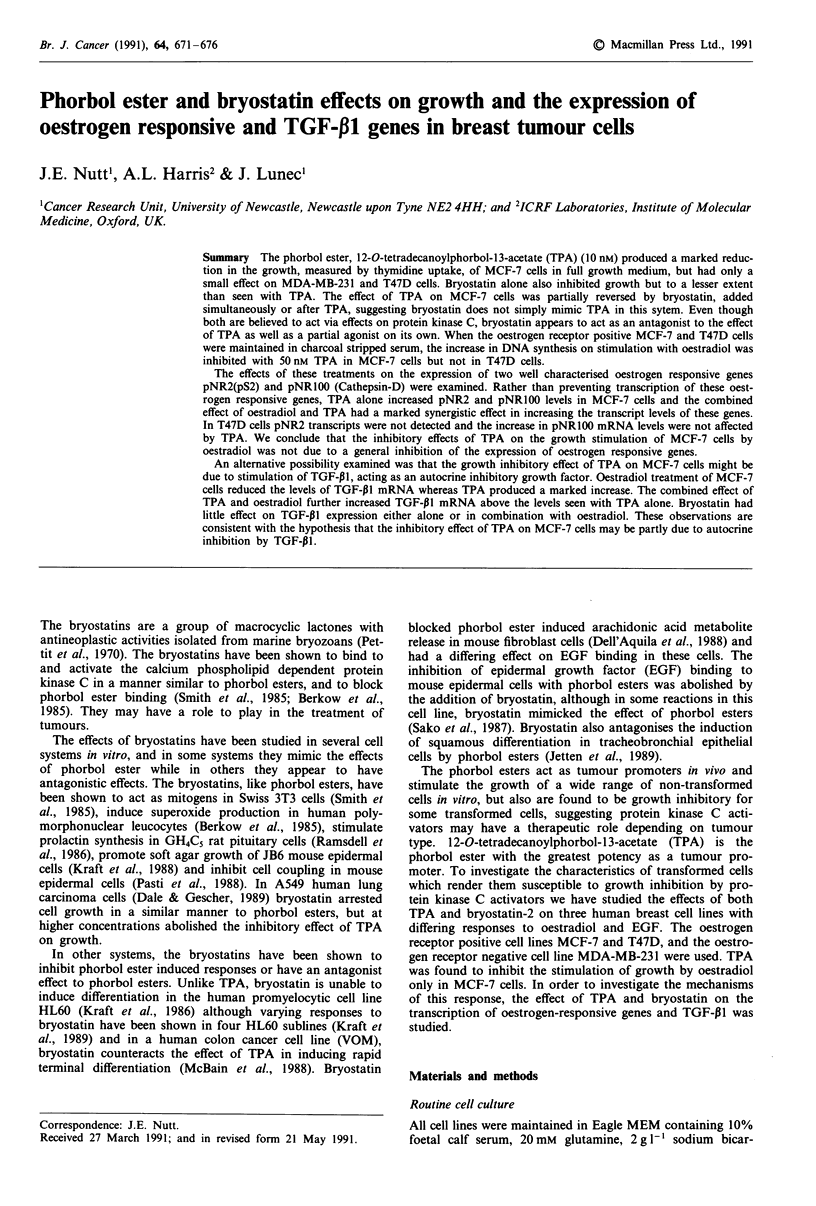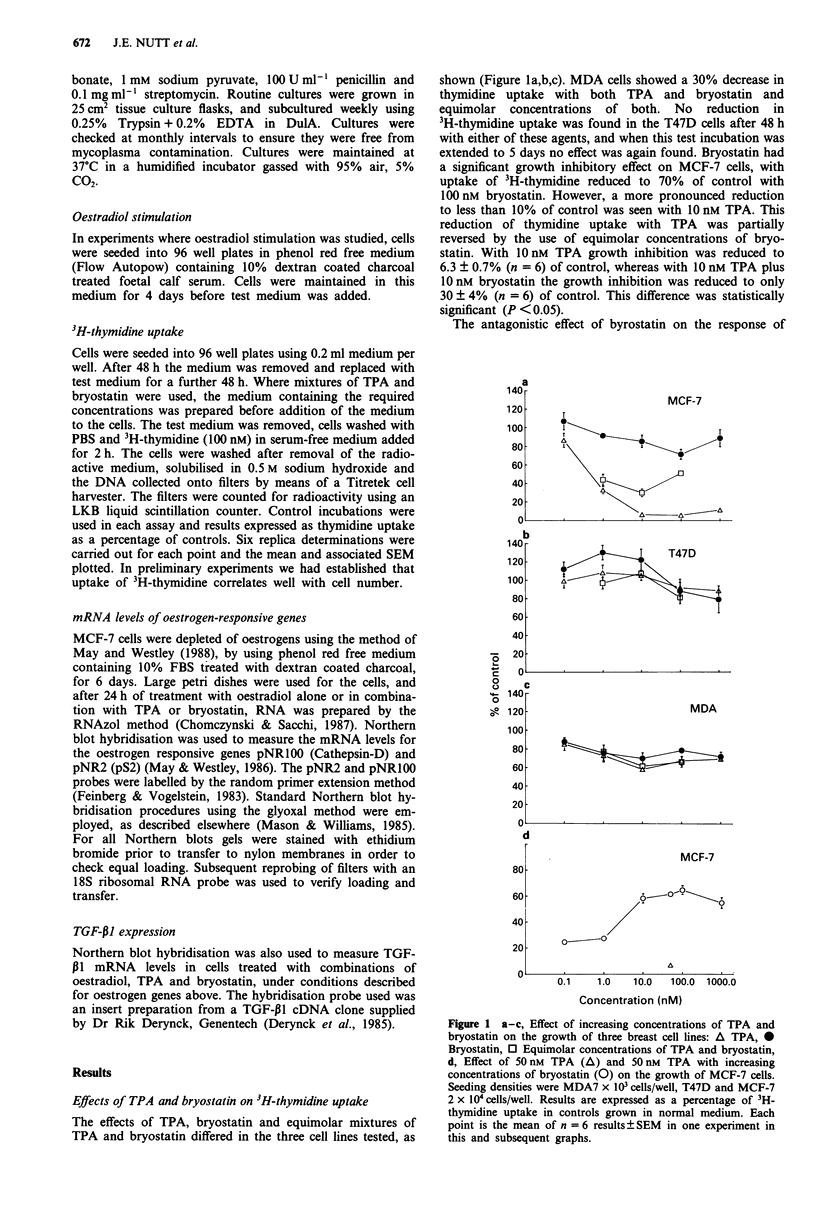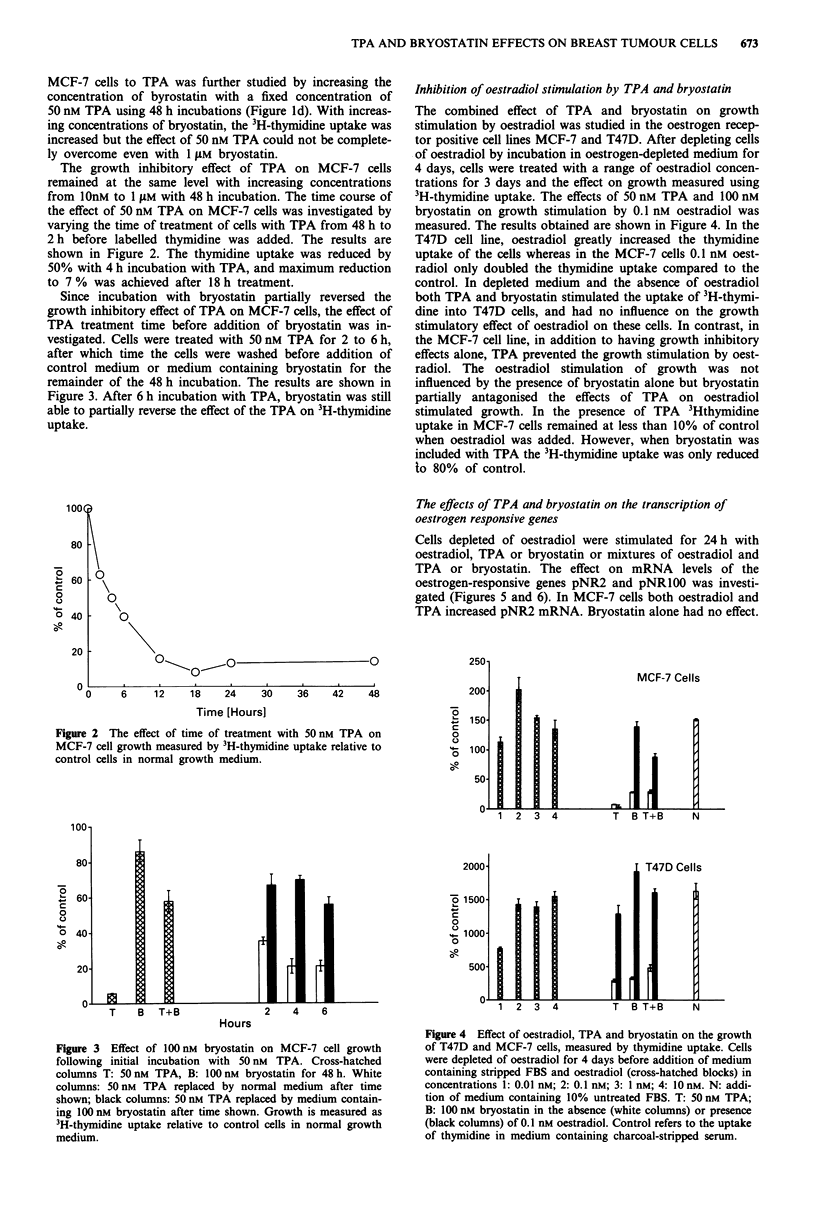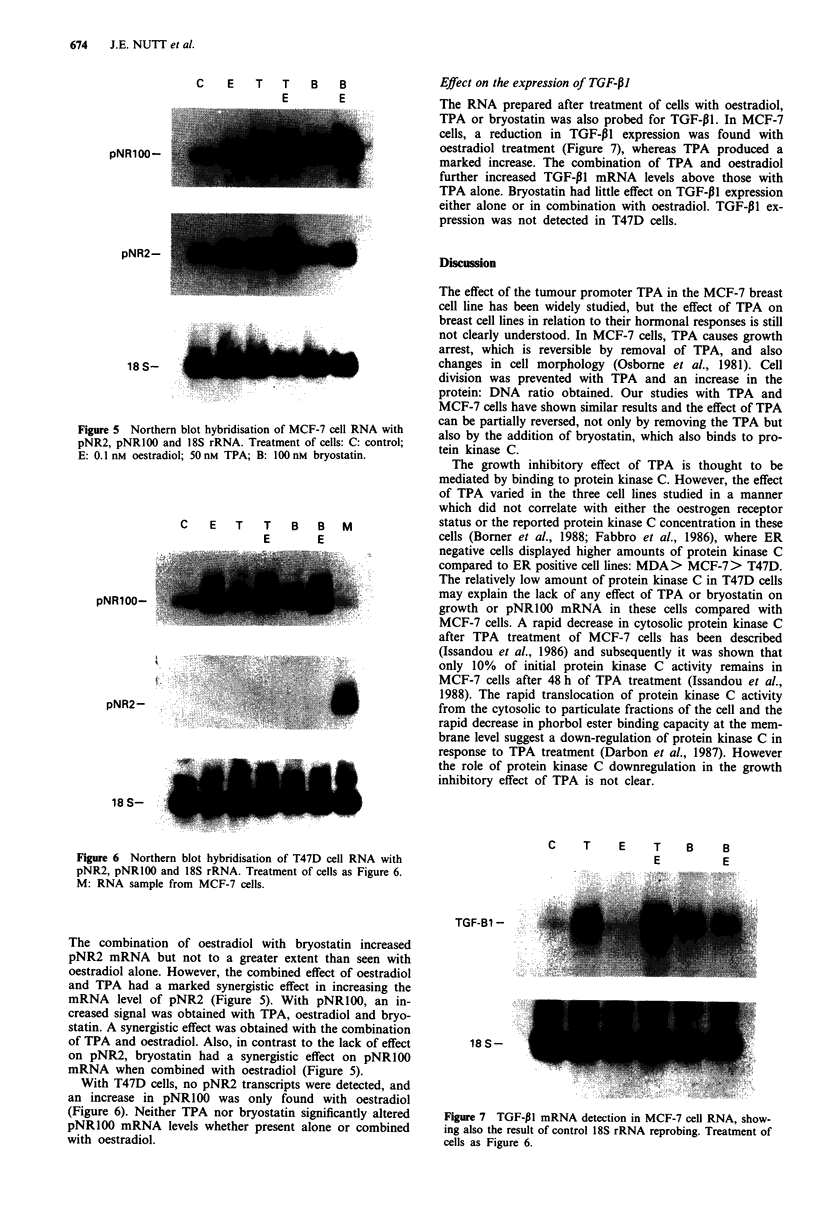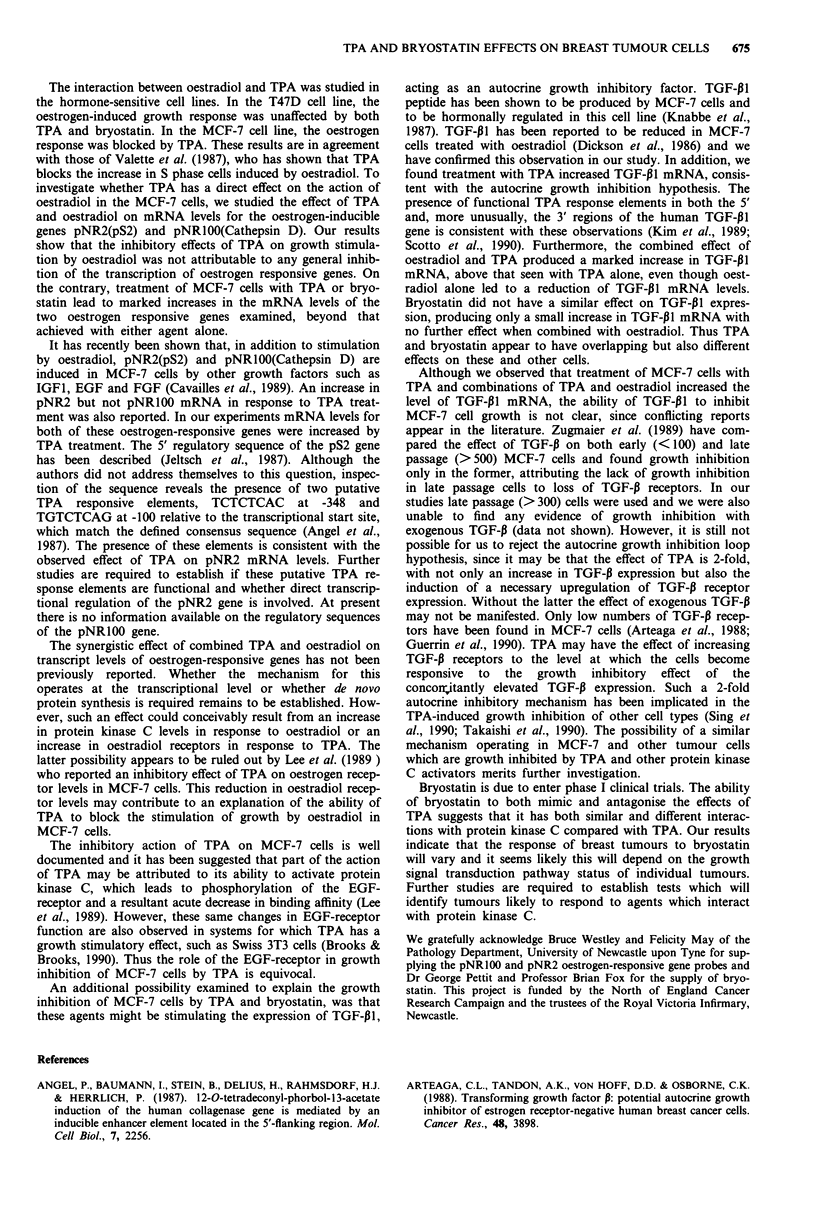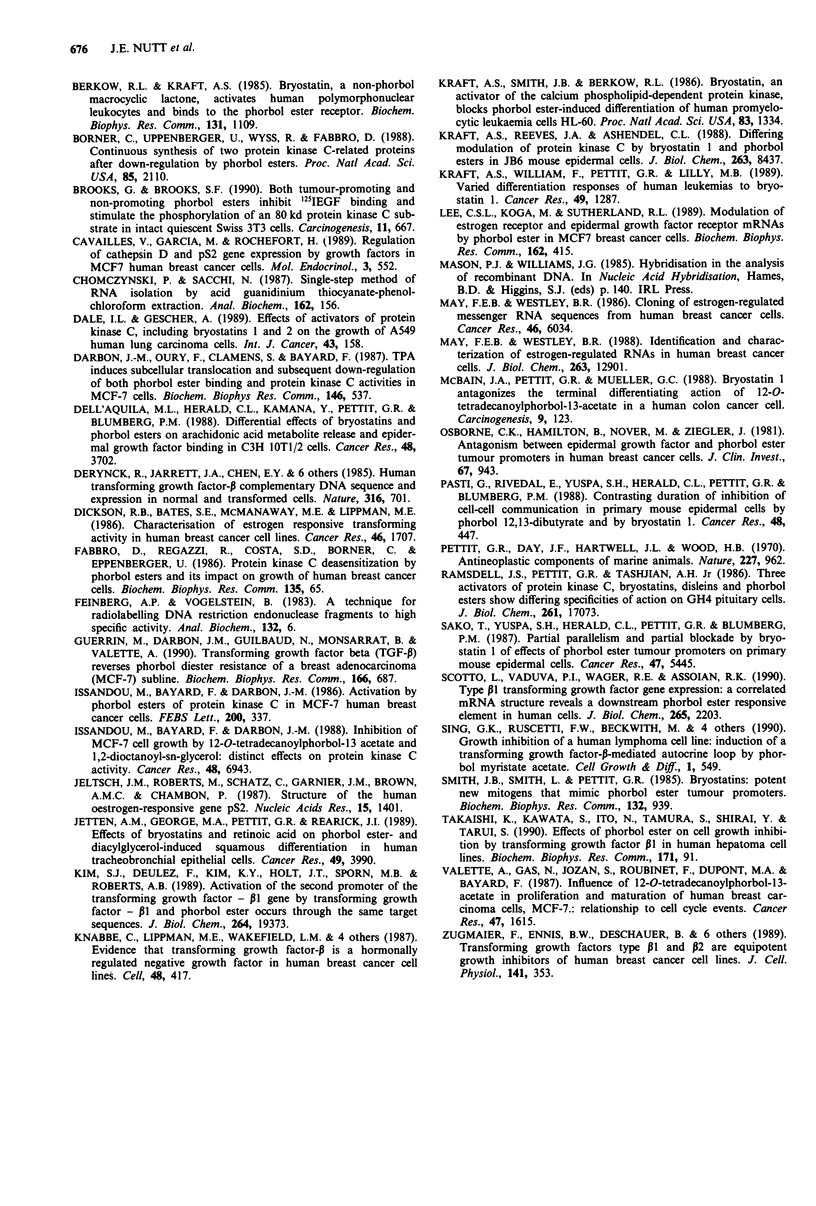# Phorbol ester and bryostatin effects on growth and the expression of oestrogen responsive and TGF-beta 1 genes in breast tumour cells.

**DOI:** 10.1038/bjc.1991.379

**Published:** 1991-10

**Authors:** J. E. Nutt, A. L. Harris, J. Lunec

**Affiliations:** Cancer Research Unit, University of Newcastle, Newcastle upon Tyne, UK.

## Abstract

**Images:**


Br. J. Cancer (1991), 64, 671-676
© Macmillan Press Ltd., 1991

### Phorbol ester and bryostatin effects on growth and the expression of oestrogen responsive and TGF-β1 genes in breast tumour cells

J.E. Nutt[1], A.L. Harris[2] & J. Lunec[1]

[1]*Cancer Research Unit, University of Newcastle, Newcastle upon Tyne NE2 4HH; and* [2]*ICRF Laboratories, Institute of Molecular Medicine, Oxford, UK.*

**Summary** The phorbol ester, 12-*O*-tetradecanoylphorbol-13-acetate (TPA) (10 nM) produced a marked reduction in the growth, measured by thymidine uptake, of MCF-7 cells in full growth medium, but had only a small effect on MDA-MB-231 and T47D cells. Bryostatin alone also inhibited growth but to a lesser extent than seen with TPA. The effect of TPA on MCF-7 cells was partially reversed by bryostatin, added simultaneously or after TPA, suggesting bryostatin does not simply mimic TPA in this sytem. Even though both are believed to act via effects on protein kinase C, bryostatin appears to act as an antagonist to the effect of TPA as well as a partial agonist on its own. When the oestrogen receptor positive MCF-7 and T47D cells were maintained in charcoal stripped serum, the increase in DNA synthesis on stimulation with oestradiol was inhibited with 50 nM TPA in MCF-7 cells but not in T47D cells.

The effects of these treatments on the expression of two well characterised oestrogen responsive genes pNR2(pS2) and pNR100 (Cathepsin-D) were examined. Rather than preventing transcription of these oestrogen responsive genes, TPA alone increased pNR2 and pNR100 levels in MCF-7 cells and the combined effect of oestradiol and TPA had a marked synergistic effect in increasing the transcript levels of these genes. In T47D cells pNR2 transcripts were not detected and the increase in pNR100 mRNA levels were not affected by TPA. We conclude that the inhibitory effects of TPA on the growth stimulation of MCF-7 cells by oestradiol was not due to a general inhibition of the expression of oestrogen responsive genes.

An alternative possibility examined was that the growth inhibitory effect of TPA on MCF-7 cells might be due to stimulation of TGF-β1, acting as an autocrine inhibitory growth factor. Oestradiol treatment of MCF-7 cells reduced the levels of TGF-β1 mRNA whereas TPA produced a marked increase. The combined effect of TPA and oestradiol further increased TGF-β1 mRNA above the levels seen with TPA alone. Bryostatin had little effect on TGF-β1 expression either alone or in combination with oestradiol. These observations are consistent with the hypothesis that the inhibitory effect of TPA on MCF-7 cells may be partly due to autocrine inhibition by TGF-β1.

The bryostatins are a group of macrocyclic lactones with antineoplastic activities isolated from marine bryozoans (Pettit *et al*., 1970). The bryostatins have been shown to bind to and activate the calcium phospholipid dependent protein kinase C in a manner similar to phorbol esters, and to block phorbol ester binding (Smith *et al*., 1985; Berkow *et al*., 1985). They may have a role to play in the treatment of tumours.

The effects of bryostatins have been studied in several cell systems *in vitro*, and in some systems they mimic the effects of phorbol ester while in others they appear to have antagonistic effects. The bryostatins, like phorbol esters, have been shown to act as mitogens in Swiss 3T3 cells (Smith *et al*., 1985), induce superoxide production in human polymorphonuclear leucocytes (Berkow *et al*., 1985), stimulate prolactin synthesis in $GH_4C_5$ rat pituitary cells (Ramsdell *et al*., 1986), promote soft agar growth of JB6 mouse epidermal cells (Kraft *et al*., 1988) and inhibit cell coupling in mouse epidermal cells (Pasti *et al*., 1988). In A549 human lung carcinoma cells (Dale & Gescher, 1989) bryostatin arrested cell growth in a similar manner to phorbol esters, but at higher concentrations abolished the inhibitory effect of TPA on growth.

In other systems, the bryostatins have been shown to inhibit phorbol ester induced responses or have an antagonist effect to phorbol esters. Unlike TPA, bryostatin is unable to induce differentiation in the human promyelocytic cell line HL60 (Kraft *et al*., 1986) although varying responses to bryostatin have been shown in four HL60 sublines (Kraft *et al*., 1989) and in a human colon cancer cell line (VOM), bryostatin counteracts the effect of TPA in inducing rapid terminal differentiation (McBain *et al*., 1988). Bryostatin blocked phorbol ester induced arachidonic acid metabolite release in mouse fibroblast cells (Dell'Aquila *et al*., 1988) and had a differing effect on EGF binding in these cells. The inhibition of epidermal growth factor (EGF) binding to mouse epidermal cells with phorbol esters was abolished by the addition of bryostatin, although in some reactions in this cell line, bryostatin mimicked the effect of phorbol esters (Sako *et al*., 1987). Bryostatin also antagonises the induction of squamous differentiation in tracheobronchial epithelial cells by phorbol esters (Jetten *et al*., 1989).

The phorbol esters act as tumour promoters *in vivo* and stimulate the growth of a wide range of non-transformed cells *in vitro*, but also are found to be growth inhibitory for some transformed cells, suggesting protein kinase C activators may have a therapeutic role depending on tumour type. 12-*O*-tetradecanoylphorbol-13-acetate (TPA) is the phorbol ester with the greatest potency as a tumour promoter. To investigate the characteristics of transformed cells which render them susceptible to growth inhibition by protein kinase C activators we have studied the effects of both TPA and bryostatin-2 on three human breast cell lines with differing responses to oestradiol and EGF. The oestrogen receptor positive cell lines MCF-7 and T47D, and the oestrogen receptor negative cell line MDA-MB-231 were used. TPA was found to inhibit the stimulation of growth by oestradiol only in MCF-7 cells. In order to investigate the mechanisms of this response, the effect of TPA and bryostatin on the transcription of oestrogen-responsive genes and TGF-β1 was studied.

### Materials and methods

### *Routine cell culture*

All cell lines were maintained in Eagle MEM containing 10% foetal calf serum, 20 mM glutamine, 2 g $l^{-1}$ sodium bicar-

Correspondence: J.E. Nutt.
Received 27 March 1991; and in revised form 21 May 1991.

bonate, 1 mM sodium pyruvate, $100\,U\,ml^{-1}$ penicillin and $0.1\,mg\,ml^{-1}$ streptomycin. Routine cultures were grown in $25\,cm^2$ tissue culture flasks, and subcultured weekly using 0.25% Trypsin + 0.2% EDTA in DulA. Cultures were checked at monthly intervals to ensure they were free from mycoplasma contamination. Cultures were maintained at 37°C in a humidified incubator gassed with 95% air, 5% $CO_2$.

### *Oestradiol stimulation*

In experiments where oestradiol stimulation was studied, cells were seeded into 96 well plates in phenol red free medium (Flow Autopow) containing 10% dextran coated charcoal treated foetal calf serum. Cells were maintained in this medium for 4 days before test medium was added.

### *$^3$H-thymidine uptake*

Cells were seeded into 96 well plates using 0.2 ml medium per well. After 48 h the medium was removed and replaced with test medium for a further 48 h. Where mixtures of TPA and bryostatin were used, the medium containing the required concentrations was prepared before addition of the medium to the cells. The test medium was removed, cells washed with PBS and $^3$H-thymidine (100 nM) in serum-free medium added for 2 h. The cells were washed after removal of the radioactive medium, solubilised in 0.5 M sodium hydroxide and the DNA collected onto filters by means of a Titretek cell harvester. The filters were counted for radioactivity using an LKB liquid scintillation counter. Control incubations were used in each assay and results expressed as thymidine uptake as a percentage of controls. Six replica determinations were carried out for each point and the mean and associated SEM plotted. In preliminary experiments we had established that uptake of $^3$H-thymidine correlates well with cell number.

### *mRNA levels of oestrogen-responsive genes*

MCF-7 cells were depleted of oestrogens using the method of May and Westley (1988), by using phenol red free medium containing 10% FBS treated with dextran coated charcoal, for 6 days. Large petri dishes were used for the cells, and after 24 h of treatment with oestradiol alone or in combination with TPA or bryostatin, RNA was prepared by the RNAzol method (Chomczynski & Sacchi, 1987). Northern blot hybridisation was used to measure the mRNA levels for the oestrogen responsive genes pNR100 (Cathepsin-D) and pNR2 (pS2) (May & Westley, 1986). The pNR2 and pNR100 probes were labelled by the random primer extension method (Feinberg & Vogelstein, 1983). Standard Northern blot hybridisation procedures using the glyoxal method were employed, as described elsewhere (Mason & Williams, 1985). For all Northern blots gels were stained with ethidium bromide prior to transfer to nylon membranes in order to check equal loading. Subsequent reprobing of filters with an 18S ribosomal RNA probe was used to verify loading and transfer.

### *TGF-β1 expression*

Northern blot hybridisation was also used to measure TGF-β1 mRNA levels in cells treated with combinations of oestradiol, TPA and bryostatin, under conditions described for oestrogen genes above. The hybridisation probe used was an insert preparation from a TGF-β1 cDNA clone supplied by Dr Rik Derynck, Genentech (Derynck *et al.*, 1985).

### Results

### *Effects of TPA and bryostatin on $^3$H-thymidine uptake*

The effects of TPA, bryostatin and equimolar mixtures of TPA and bryostatin differed in the three cell lines tested, as shown (Figure 1a,b,c). MDA cells showed a 30% decrease in thymidine uptake with both TPA and bryostatin and equimolar concentrations of both. No reduction in $^3$H-thymidine uptake was found in the T47D cells after 48 h with either of these agents, and when this test incubation was extended to 5 days no effect was again found. Bryostatin had a significant growth inhibitory effect on MCF-7 cells, with uptake of $^3$H-thymidine reduced to 70% of control with 100 nM bryostatin. However, a more pronounced reduction to less than 10% of control was seen with 10 nM TPA. This reduction of thymidine uptake with TPA was partially reversed by the use of equimolar concentrations of bryostatin. With 10 nM TPA growth inhibition was reduced to $6.3 \pm 0.7\%$ ($n = 6$) of control, whereas with 10 nM TPA plus 10 nM bryostatin the growth inhibition was reduced to only $30 \pm 4\%$ ($n = 6$) of control. This difference was statistically significant ($P < 0.05$).

The antagonistic effect of byrostatin on the response of

**Figure 1** a–c, Effect of increasing concentrations of TPA and bryostatin on the growth of three breast cell lines: Δ TPA, ● Bryostatin, □ Equimolar concentrations of TPA and bryostatin, d, Effect of 50 nM TPA (Δ) and 50 nM TPA with increasing concentrations of bryostatin (O) on the growth of MCF-7 cells. Seeding densities were MDA7 × $10^3$ cells/well, T47D and MCF-7 2 × $10^4$ cells/well. Results are expressed as a percentage of $^3$H-thymidine uptake in controls grown in normal medium. Each point is the mean of $n = 6$ results ± SEM in one experiment in this and subsequent graphs.

MCF-7 cells to TPA was further studied by increasing the concentration of byrostatin with a fixed concentration of 50 nM TPA using 48 h incubations (Figure 1d). With increasing concentrations of bryostatin, the $^{3}$H-thymidine uptake was increased but the effect of 50 nM TPA could not be completely overcome even with 1 μM bryostatin.

The growth inhibitory effect of TPA on MCF-7 cells remained at the same level with increasing concentrations from 10nM to 1 μM with 48 h incubation. The time course of the effect of 50 nM TPA on MCF-7 cells was investigated by varying the time of treatment of cells with TPA from 48 h to 2 h before labelled thymidine was added. The results are shown in Figure 2. The thymidine uptake was reduced by 50% with 4 h incubation with TPA, and maximum reduction to 7 % was achieved after 18 h treatment.

Since incubation with bryostatin partially reversed the growth inhibitory effect of TPA on MCF-7 cells, the effect of TPA treatment time before addition of bryostatin was investigated. Cells were treated with 50 nM TPA for 2 to 6 h, after which time the cells were washed before addition of control medium or medium containing bryostatin for the remainder of the 48 h incubation. The results are shown in Figure 3. After 6 h incubation with TPA, bryostatin was still able to partially reverse the effect of the TPA on $^{3}$H-thymidine uptake.

**Figure 2** The effect of time of treatment with 50 nM TPA on MCF-7 cell growth measured by $^{3}$H-thymidine uptake relative to control cells in normal growth medium.

**Figure 3** Effect of 100 nM bryostatin on MCF-7 cell growth following initial incubation with 50 nM TPA. Cross-hatched columns T: 50 nM TPA, B: 100 nM bryostatin for 48 h. White columns: 50 nM TPA replaced by normal medium after time shown; black columns: 50 nM TPA replaced by medium containing 100 nM bryostatin after time shown. Growth is measured as $^{3}$H-thymidine uptake relative to control cells in normal growth medium.

*Inhibition of oestradiol stimulation by TPA and bryostatin*

The combined effect of TPA and bryostatin on growth stimulation by oestradiol was studied in the oestrogen receptor positive cell lines MCF-7 and T47D. After depleting cells of oestradiol by incubation in oestrogen-depleted medium for 4 days, cells were treated with a range of oestradiol concentrations for 3 days and the effect on growth measured using $^{3}$H-thymidine uptake. The effects of 50 nM TPA and 100 nM bryostatin on growth stimulation by 0.1 nM oestradiol was measured. The results obtained are shown in Figure 4. In the T47D cell line, oestradiol greatly increased the thymidine uptake of the cells whereas in the MCF-7 cells 0.1 nM oestradiol only doubled the thymidine uptake compared to the control. In depleted medium and the absence of oestradiol both TPA and bryostatin stimulated the uptake of $^{3}$H-thymidine into T47D cells, and had no influence on the growth stimulatory effect of oestradiol on these cells. In contrast, in the MCF-7 cell line, in addition to having growth inhibitory effects alone, TPA prevented the growth stimulation by oestradiol. The oestradiol stimulation of growth was not influenced by the presence of bryostatin alone but bryostatin partially antagonised the effects of TPA on oestradiol stimulated growth. In the presence of TPA $^{3}$Hthymidine uptake in MCF-7 cells remained at less than 10% of control when oestradiol was added. However, when bryostatin was included with TPA the $^{3}$H-thymidine uptake was only reduced to 80% of control.

*The effects of TPA and bryostatin on the transcription of oestrogen responsive genes*

Cells depleted of oestradiol were stimulated for 24 h with oestradiol, TPA or bryostatin or mixtures of oestradiol and TPA or bryostatin. The effect on mRNA levels of the oestrogen-responsive genes pNR2 and pNR100 was investigated (Figures 5 and 6). In MCF-7 cells both oestradiol and TPA increased pNR2 mRNA. Bryostatin alone had no effect.

**Figure 4** Effect of oestradiol, TPA and bryostatin on the growth of T47D and MCF-7 cells, measured by thymidine uptake. Cells were depleted of oestradiol for 4 days before addition of medium containing stripped FBS and oestradiol (cross-hatched blocks) in concentrations 1: 0.01 nM; 2: 0.1 nM; 3: 1 nM; 4: 10 nM. N: addition of medium containing 10% untreated FBS. T: 50 nM TPA; B: 100 nM bryostatin in the absence (white columns) or presence (black columns) of 0.1 nM oestradiol. Control refers to the uptake of thymidine in medium containing charcoal-stripped serum.

**Figure 5** Northern blot hybridisation of MCF-7 cell RNA with pNR2, pNR100 and 18S rRNA. Treatment of cells: C: control; E: 0.1 nM oestradiol; 50 nM TPA; B: 100 nM bryostatin.

**Figure 6** Northern blot hybridisation of T47D cell RNA with pNR2, pNR100 and 18S rRNA. Treatment of cells as Figure 6. M: RNA sample from MCF-7 cells.

The combination of oestradiol with bryostatin increased pNR2 mRNA but not to a greater extent than seen with oestradiol alone. However, the combined effect of oestradiol and TPA had a marked synergistic effect in increasing the mRNA level of pNR2 (Figure 5). With pNR100, an increased signal was obtained with TPA, oestradiol and bryostatin. A synergistic effect was obtained with the combination of TPA and oestradiol. Also, in contrast to the lack of effect on pNR2, bryostatin had a synergistic effect on pNR100 mRNA when combined with oestradiol (Figure 5).

With T47D cells, no pNR2 transcripts were detected, and an increase in pNR100 was only found with oestradiol (Figure 6). Neither TPA nor bryostatin significantly altered pNR100 mRNA levels whether present alone or combined with oestradiol.

### *Effect on the expression of TGF-β1*

The RNA prepared after treatment of cells with oestradiol, TPA or bryostatin was also probed for TGF-β1. In MCF-7 cells, a reduction in TGF-β1 expression was found with oestradiol treatment (Figure 7), whereas TPA produced a marked increase. The combination of TPA and oestradiol further increased TGF-β1 mRNA levels above those with TPA alone. Bryostatin had little effect on TGF-β1 expression either alone or in combination with oestradiol. TGF-β1 expression was not detected in T47D cells.

### Discussion

The effect of the tumour promoter TPA in the MCF-7 breast cell line has been widely studied, but the effect of TPA on breast cell lines in relation to their hormonal responses is still not clearly understood. In MCF-7 cells, TPA causes growth arrest, which is reversible by removal of TPA, and also changes in cell morphology (Osborne *et al.*, 1981). Cell division was prevented with TPA and an increase in the protein: DNA ratio obtained. Our studies with TPA and MCF-7 cells have shown similar results and the effect of TPA can be partially reversed, not only by removing the TPA but also by the addition of bryostatin, which also binds to protein kinase C.

The growth inhibitory effect of TPA is thought to be mediated by binding to protein kinase C. However, the effect of TPA varied in the three cell lines studied in a manner which did not correlate with either the oestrogen receptor status or the reported protein kinase C concentration in these cells (Borner *et al.*, 1988; Fabbro *et al.*, 1986), where ER negative cells displayed higher amounts of protein kinase C compared to ER positive cell lines: MDA > MCF-7 > T47D. The relatively low amount of protein kinase C in T47D cells may explain the lack of any effect of TPA or bryostatin on growth or pNR100 mRNA in these cells compared with MCF-7 cells. A rapid decrease in cytosolic protein kinase C after TPA treatment of MCF-7 cells has been described (Issandou *et al.*, 1986) and subsequently it was shown that only 10% of initial protein kinase C activity remains in MCF-7 cells after 48 h of TPA treatment (Issandou *et al.*, 1988). The rapid translocation of protein kinase C activity from the cytosolic to particulate fractions of the cell and the rapid decrease in phorbol ester binding capacity at the membrane level suggest a down-regulation of protein kinase C in response to TPA treatment (Darbon *et al.*, 1987). However the role of protein kinase C downregulation in the growth inhibitory effect of TPA is not clear.

**Figure 7** TGF-β1 mRNA detection in MCF-7 cell RNA, showing also the result of control 18S rRNA reprobing. Treatment of cells as Figure 6.

The interaction between oestradiol and TPA was studied in the hormone-sensitive cell lines. In the T47D cell line, the oestrogen-induced growth response was unaffected by both TPA and bryostatin. In the MCF-7 cell line, the oestrogen response was blocked by TPA. These results are in agreement with those of Valette *et al.* (1987), who has shown that TPA blocks the increase in S phase cells induced by oestradiol. To investigate whether TPA has a direct effect on the action of oestradiol in the MCF-7 cells, we studied the effect of TPA and oestradiol on mRNA levels for the oestrogen-inducible genes pNR2(pS2) and pNR100(Cathepsin D). Our results show that the inhibitory effects of TPA on growth stimulation by oestradiol was not attributable to any general inhibition of the transcription of oestrogen responsive genes. On the contrary, treatment of MCF-7 cells with TPA or bryostatin lead to marked increases in the mRNA levels of the two oestrogen responsive genes examined, beyond that achieved with either agent alone.

It has recently been shown that, in addition to stimulation by oestradiol, pNR2(pS2) and pNR100(Cathepsin D) are induced in MCF-7 cells by other growth factors such as IGF1, EGF and FGF (Cavailles *et al.*, 1989). An increase in pNR2 but not pNR100 mRNA in response to TPA treatment was also reported. In our experiments mRNA levels for both of these oestrogen-responsive genes were increased by TPA treatment. The 5′ regulatory sequence of the pS2 gene has been described (Jeltsch *et al.*, 1987). Although the authors did not address themselves to this question, inspection of the sequence reveals the presence of two putative TPA responsive elements, TCTCTCAC at -348 and TGTCTCAG at -100 relative to the transcriptional start site, which match the defined consensus sequence (Angel *et al.*, 1987). The presence of these elements is consistent with the observed effect of TPA on pNR2 mRNA levels. Further studies are required to establish if these putative TPA response elements are functional and whether direct transcriptional regulation of the pNR2 gene is involved. At present there is no information available on the regulatory sequences of the pNR100 gene.

The synergistic effect of combined TPA and oestradiol on transcript levels of oestrogen-responsive genes has not been previously reported. Whether the mechanism for this operates at the transcriptional level or whether *de novo* protein synthesis is required remains to be established. However, such an effect could conceivably result from an increase in protein kinase C levels in response to oestradiol or an increase in oestradiol receptors in response to TPA. The latter possibility appears to be ruled out by Lee *et al.* (1989 ) who reported an inhibitory effect of TPA on oestrogen receptor levels in MCF-7 cells. This reduction in oestradiol receptor levels may contribute to an explanation of the ability of TPA to block the stimulation of growth by oestradiol in MCF-7 cells.

The inhibitory action of TPA on MCF-7 cells is well documented and it has been suggested that part of the action of TPA may be attributed to its ability to activate protein kinase C, which leads to phosphorylation of the EGF-receptor and a resultant acute decrease in binding affinity (Lee *et al.*, 1989). However, these same changes in EGF-receptor function are also observed in systems for which TPA has a growth stimulatory effect, such as Swiss 3T3 cells (Brooks & Brooks, 1990). Thus the role of the EGF-receptor in growth inhibition of MCF-7 cells by TPA is equivocal.

An additional possibility examined to explain the growth inhibition of MCF-7 cells by TPA and bryostatin, was that these agents might be stimulating the expression of TGF-$\beta$1, acting as an autocrine growth inhibitory factor. TGF-$\beta$1 peptide has been shown to be produced by MCF-7 cells and to be hormonally regulated in this cell line (Knabbe *et al.*, 1987). TGF-$\beta$1 has been reported to be reduced in MCF-7 cells treated with oestradiol (Dickson *et al.*, 1986) and we have confirmed this observation in our study. In addition, we found treatment with TPA increased TGF-$\beta$1 mRNA, consistent with the autocrine growth inhibition hypothesis. The presence of functional TPA response elements in both the 5′ and, more unusually, the 3′ regions of the human TGF-$\beta$1 gene is consistent with these observations (Kim *et al.*, 1989; Scotto *et al.*, 1990). Furthermore, the combined effect of oestradiol and TPA produced a marked increase in TGF-$\beta$1 mRNA, above that seen with TPA alone, even though oestradiol alone led to a reduction of TGF-$\beta$1 mRNA levels. Bryostatin did not have a similar effect on TGF-$\beta$1 expression, producing only a small increase in TGF-$\beta$1 mRNA with no further effect when combined with oestradiol. Thus TPA and bryostatin appear to have overlapping but also different effects on these and other cells.

Although we observed that treatment of MCF-7 cells with TPA and combinations of TPA and oestradiol increased the level of TGF-$\beta$1 mRNA, the ability of TGF-$\beta$1 to inhibit MCF-7 cell growth is not clear, since conflicting reports appear in the literature. Zugmaier *et al.* (1989) have compared the effect of TGF-$\beta$ on both early ($<100$) and late passage ($>500$) MCF-7 cells and found growth inhibition only in the former, attributing the lack of growth inhibition in late passage cells to loss of TGF-$\beta$ receptors. In our studies late passage ($>300$) cells were used and we were also unable to find any evidence of growth inhibition with exogenous TGF-$\beta$ (data not shown). However, it is still not possible for us to reject the autocrine growth inhibition loop hypothesis, since it may be that the effect of TPA is 2-fold, with not only an increase in TGF-$\beta$ expression but also the induction of a necessary upregulation of TGF-$\beta$ receptor expression. Without the latter the effect of exogenous TGF-$\beta$ may not be manifested. Only low numbers of TGF-$\beta$ receptors have been found in MCF-7 cells (Arteaga *et al.*, 1988; Guerrin *et al.*, 1990). TPA may have the effect of increasing TGF-$\beta$ receptors to the level at which the cells become responsive to the growth inhibitory effect of the concomitantly elevated TGF-$\beta$ expression. Such a 2-fold autocrine inhibitory mechanism has been implicated in the TPA-induced growth inhibition of other cell types (Sing *et al.*, 1990; Takaishi *et al.*, 1990). The possibility of a similar mechanism operating in MCF-7 and other tumour cells which are growth inhibited by TPA and other protein kinase C activators merits further investigation.

Bryostatin is due to enter phase I clinical trials. The ability of bryostatin to both mimic and antagonise the effects of TPA suggests that it has both similar and different interactions with protein kinase C compared with TPA. Our results indicate that the response of breast tumours to bryostatin will vary and it seems likely this will depend on the growth signal transduction pathway status of individual tumours. Further studies are required to establish tests which will identify tumours likely to respond to agents which interact with protein kinase C.

We gratefully acknowledge Bruce Westley and Felicity May of the Pathology Department, University of Newcastle upon Tyne for supplying the pNR100 and pNR2 oestrogen-responsive gene probes and Dr George Pettit and Professor Brian Fox for the supply of bryostatin. This project is funded by the North of England Cancer Research Campaign and the trustees of the Royal Victoria Infirmary, Newcastle.

### References

ANGEL, P., BAUMANN, I., STEIN, B., DELIUS, H., RAHMSDORF, H.J. & HERRLICH, P. (1987). 12-*O*-tetradeconyl-phorbol-13-acetate induction of the human collagenase gene is mediated by an inducible enhancer element located in the 5′-flanking region. *Mol. Cell Biol.*, **7**, 2256.

ARTEAGA, C.L., TANDON, A.K., VON HOFF, D.D. & OSBORNE, C.K. (1988). Transforming growth factor $\beta$: potential autocrine growth inhibitor of estrogen receptor-negative human breast cancer cells. *Cancer Res.*, **48**, 3898.

BERKOW, R.L. & KRAFT, A.S. (1985). Bryostatin, a non-phorbol macrocyclic lactone, activates human polymorphonuclear leukocytes and binds to the phorbol ester receptor. *Biochem. Biophys. Res. Comm.*, **131**, 1109.

BORNER, C., UPPENBERGER, U., WYSS, R. & FABBRO, D. (1988). Continuous synthesis of two protein kinase C-related proteins after down-regulation by phorbol esters. *Proc. Natl Acad. Sci. USA*, **85**, 2110.

BROOKS, G. & BROOKS, S.F. (1990). Both tumour-promoting and non-promoting phorbol esters inhibit $^{125}$IEGF binding and stimulate the phosphorylation of an 80 kd protein kinase C substrate in intact quiescent Swiss 3T3 cells. *Carcinogenesis*, **11**, 667.

CAVAILLES, V., GARCIA, M. & ROCHEFORT, H. (1989). Regulation of cathepsin D and pS2 gene expression by growth factors in MCF7 human breast cancer cells. *Mol. Endocrinol.*, **3**, 552.

CHOMCZYNSKI, P. & SACCHI, N. (1987). Single-step method of RNA isolation by acid guanidinium thiocyanate-phenol-chloroform extraction. *Anal. Biochem.*, **162**, 156.

DALE, I.L. & GESCHER, A. (1989). Effects of activators of protein kinase C, including bryostatins 1 and 2 on the growth of A549 human lung carcinoma cells. *Int. J. Cancer*, **43**, 158.

DARBON, J.-M., OURY, F., CLAMENS, S. & BAYARD, F. (1987). TPA induces subcellular translocation and subsequent down-regulation of both phorbol ester binding and protein kinase C activities in MCF-7 cells. *Biochem. Biophys Res. Comm.*, **146**, 537.

DELL'AQUILA, M.L., HERALD, C.L., KAMANA, Y., PETTIT, G.R. & BLUMBERG, P.M. (1988). Differential effects of bryostatins and phorbol esters on arachidonic acid metabolite release and epidermal growth factor binding in C3H 10T1/2 cells. *Cancer Res.*, **48**, 3702.

DERYNCK, R., JARRETT, J.A., CHEN, E.Y. & 6 others (1985). Human transforming growth factor-$\beta$ complementary DNA sequence and expression in normal and transformed cells. *Nature*, **316**, 701.

DICKSON, R.B., BATES, S.E., MCMANAWAY, M.E. & LIPPMAN, M.E. (1986). Characterisation of estrogen responsive transforming activity in human breast cancer cell lines. *Cancer Res.*, **46**, 1707.

FABBRO, D., REGAZZI, R., COSTA, S.D., BORNER, C. & EPPENBERGER, U. (1986). Protein kinase C deasensitization by phorbol esters and its impact on growth of human breast cancer cells. *Biochem. Biophys. Res. Comm* **135**, 65.

FEINBERG, A.P. & VOGELSTEIN, B. (1983). A technique for radiolabelling DNA restriction endonuclease fragments to high specific activity. *Anal. Biochem.*, **132**, 6.

GUERRIN, M., DARBON, J.M., GUILBAUD, N., MONSARRAT, B. & VALETTE, A. (1990). Transforming growth factor beta (TGF-$\beta$) reverses phorbol diester resistance of a breast adenocarcinoma (MCF-7) subline. *Biochem. Biophys. Res. Comm.*, **166**, 687.

ISSANDOU, M., BAYARD, F. & DARBON, J.-M. (1986). Activation by phorbol esters of protein kinase C in MCF-7 human breast cancer cells. *FEBS Lett.*, **200**, 337.

ISSANDOU, M., BAYARD, F. & DARBON, J.-M. (1988). Inhibition of MCF-7 cell growth by 12-*O*-tetradecanoylphorbol-13 acetate and 1,2-dioctanoyl-sn-glycerol: distinct effects on protein kinase C activity. *Cancer Res.*, **48**, 6943.

JELTSCH, J.M., ROBERTS, M., SCHATZ, C., GARNIER, J.M., BROWN, A.M.C. & CHAMBON, P. (1987). Structure of the human oestrogen-responsive gene pS2. *Nucleic Acids Res.*, **15**, 1401.

JETTEN, A.M., GEORGE, M.A., PETTIT, G.R. & REARICK, J.I. (1989). Effects of bryostatins and retinoic acid on phorbol ester- and diacylglycerol-induced squamous differentiation in human tracheobronchial epithelial cells. *Cancer Res.*, **49**, 3990.

KIM, S.J., DEULEZ, F., KIM, K.Y., HOLT, J.T., SPORN, M.B. & ROBERTS, A.B. (1989). Activation of the second promoter of the transforming growth factor – $\beta$1 gene by transforming growth factor – $\beta$1 and phorbol ester occurs through the same target sequences. *J. Biol. Chem.*, **264**, 19373.

KNABBE, C., LIPPMAN, M.E., WAKEFIELD, L.M. & 4 others (1987). Evidence that transforming growth factor-$\beta$ is a hormonally regulated negative growth factor in human breast cancer cell lines. *Cell*, **48**, 417.

KRAFT, A.S., SMITH, J.B. & BERKOW, R.L. (1986). Bryostatin, an activator of the calcium phospholipid-dependent protein kinase, blocks phorbol ester-induced differentiation of human promyelocytic leukaemia cells HL-60. *Proc. Natl Acad. Sci. USA*, **83**, 1334.

KRAFT, A.S., REEVES, J.A. & ASHENDEL, C.L. (1988). Differing modulation of protein kinase C by bryostatin 1 and phorbol esters in JB6 mouse epidermal cells. *J. Biol. Chem.*, **263**, 8437.

KRAFT, A.S., WILLIAM, F., PETTIT, G.R. & LILLY, M.B. (1989). Varied differentiation responses of human leukemias to bryostatin 1. *Cancer Res.*, **49**, 1287.

LEE, C.S.L., KOGA, M. & SUTHERLAND, R.L. (1989). Modulation of estrogen receptor and epidermal growth factor receptor mRNAs by phorbol ester in MCF7 breast cancer cells. *Biochem. Biophys. Res. Comm.*, **162**, 415.

MASON, P.J. & WILLIAMS, J.G. (1985). Hybridisation in the analysis of recombinant DNA. In *Nucleic Acid Hybridisation*, Hames, B.D. & Higgins, S.J. (eds) p. 140. IRL Press.

MAY, F.E.B. & WESTLEY, B.R. (1986). Cloning of estrogen-regulated messenger RNA sequences from human breast cancer cells. *Cancer Res.*, **46**, 6034.

MAY, F.E.B. & WESTLEY, B.R. (1988). Identification and characterization of estrogen-regulated RNAs in human breast cancer cells. *J. Biol. Chem.*, **263**, 12901.

MCBAIN, J.A., PETTIT, G.R. & MUELLER, G.C. (1988). Bryostatin 1 antagonizes the terminal differentiating action of 12-*O*-tetradecanoylphorbol-13-acetate in a human colon cancer cell. *Carcinogenesis*, **9**, 123.

OSBORNE, C.K., HAMILTON, B., NOVER, M. & ZIEGLER, J. (1981). Antagonism between epidermal growth factor and phorbol ester tumour promoters in human breast cancer cells. *J. Clin. Invest.*, **67**, 943.

PASTI, G., RIVEDAL, E., YUSPA, S.H., HERALD, C.L., PETTIT, G.R. & BLUMBERG, P.M. (1988). Contrasting duration of inhibition of cell-cell communication in primary mouse epidermal cells by phorbol 12,13-dibutyrate and by bryostatin 1. *Cancer Res.*, **48**, 447.

PETTIT, G.R., DAY, J.F., HARTWELL, J.L. & WOOD, H.B. (1970). Antineoplastic components of marine animals. *Nature*, **227**, 962.

RAMSDELL, J.S., PETTIT, G.R. & TASHJIAN, A.H. Jr (1986). Three activators of protein kinase C, bryostatins, disleins and phorbol esters show differing specificities of action on GH4 pituitary cells. *J. Biol. Chem.*, **261**, 17073.

SAKO, T., YUSPA, S.H., HERALD, C.L., PETTIT, G.R. & BLUMBERG, P.M. (1987). Partial parallelism and partial blockade by bryostatin 1 of effects of phorbol ester tumour promoters on primary mouse epidermal cells. *Cancer Res.*, **47**, 5445.

SCOTTO, L., VADUVA, P.I., WAGER, R.E. & ASSOIAN, R.K. (1990). Type $\beta$1 transforming growth factor gene expression: a correlated mRNA structure reveals a downstream phorbol ester responsive element in human cells. *J. Biol. Chem.*, **265**, 2203.

SING, G.K., RUSCETTI, F.W., BECKWITH, M. & 4 others (1990). Growth inhibition of a human lymphoma cell line: induction of a transforming growth factor-$\beta$-mediated autocrine loop by phorbol myristate acetate. *Cell Growth & Diff.*, **1**, 549.

SMITH, J.B., SMITH, L. & PETTIT, G.R. (1985). Bryostatins: potent new mitogens that mimic phorbol ester tumour promoters. *Biochem. Biophys. Res. Comm.*, **132**, 939.

TAKAISHI, K., KAWATA, S., ITO, N., TAMURA, S., SHIRAI, Y. & TARUI, S. (1990). Effects of phorbol ester on cell growth inhibition by transforming growth factor $\beta$1 in human hepatoma cell lines. *Biochem. Biophys. Res. Comm.*, **171**, 91.

VALETTE, A., GAS, N., JOZAN, S., ROUBINET, F., DUPONT, M.A. & BAYARD, F. (1987). Influence of 12-*O*-tetradecanoylphorbol-13-acetate in proliferation and maturation of human breast carcinoma cells, MCF-7.: relationship to cell cycle events. *Cancer Res.*, **47**, 1615.

ZUGMAIER, F., ENNIS, B.W., DESCHAUER, B. & 6 others (1989). Transforming growth factors type $\beta$1 and $\beta$2 are equipotent growth inhibitors of human breast cancer cell lines. *J. Cell. Physiol.*, **141**, 353.